# The complete mitochondrial genome of *Cheilinus oxycephalus* (Perciformes: Labridae)

**DOI:** 10.1080/23802359.2019.1681913

**Published:** 2019-10-25

**Authors:** Liang Guo, Nan Zhang, Ke-Cheng Zhu, Hua-Yang Guo, Bao-Suo Liu, Dian-Chang Zhang

**Affiliations:** a Key Laboratory of South China Sea Fishery Resources Exploitation and Utilization, Ministry of Agriculture and Rural Affairs, South China Sea Fisheries Research Institute, Chinese Academy of Fishery Sciences, Guangzhou, Guangdong, China;; b Guangdong Provincial Engineer Technology Research Center, Marine Biological Seed Industry, Guangzhou, Guangdong, China

**Keywords:** *Cheilinus oxycephalus*, complete mitochondrial genome sequence

## Abstract

*Cheilinus oxycephalus* is a marine fish species, belonging to the family Labridae and naturally distributed in Indo-Pacific waters. We obtained the first complete mitochondrial genome of this species using next-generation sequencing technology. This genome is 17,698 bp, and contains 13 protein-coding genes, 2 rRNAs, 22 tRNAs, and a D-loop region. These genes are mostly encoded on the heavy strand except for *ND6* and eight tRNA genes. The AT content is 52.32%. These characteristics are similar to other bony fish. This mitochondrial genome would be used as a resource for phylogenetic reconstruction.

*Cheilinus oxycephalus* is a marine fish species, belonging to the family Labridae, naturally distributed in the tropical sea of Indo-Pacific waters and found in coral-rich areas of lagoon and seaward reefs (Froese and Pauly 2011). Coral reef biology is our research focus because of global climate change. However, there is little genome information from coral fish on this subject. In this study, we sampled a *C. oxycephalus* from Xincun Port (N 18°42′, E 109°97′) in Hainan Province, China. The muscle (Accession No.: Scsfri-Cox1800110) was preserved in absolute alcohol in Tropical and Subtropical Marine Life Museum of South China Sea Fisheries Research Institute. The DNA was extracted, and paired-end 150 bp library was constructed and sequenced on an Illumina platform. The complete mitochondrial genome was obtained with reference-guided strategy and submitted to NCBI database (Accession No.: MN399860). This is the first assembly mitochondria genome of this species. The assembly is 17,698 bp in length and contains 13 protein-coding genes, 2 rRNAs, 22 tRNAs, and a D-loop region. 13 protein-coding genes, 2 rRNAs, and 14 tRNAs are spread on heavy strands and other genes on light strand. The start codon of most protein-coding genes is ATG, except that the start codon is GTG for COXI. The AT content is 52.32%. The most common terminal codon is TAA (found in genes *ND1*, *COXI*, *ATP8*, *ATP6*, *COXIII*, *ND4L*, *ND4*, *ND5*, and *ND6*), then is the incomplete terminal codon T– (found in genes *COXII*, *ND3*, and *Cytb*), and then the TAG (*ND2*). Compared with the mitochondrial genome (GenBank: MH688049.1) of *Cheilinus undulates*, the order of *rRNA-His* (GTG) and *tRNA-Ser* (GCT) is reverse. We retrieved complete mitochondrial genome of *Cheilinus undulates*, *Cheilinus fasciatus*, *Epinephelus akaara* and *Arothron firmamentum* from NCBI database and constructed a neighbor-joining phylogenic tree using MEGA 6.0 (Tamura et al. 2013). The result supports that *C. oxycephalus* is closer related to *C. fasciatus* than *C. undulates* (Figure 1). We also searched the barcode of life data system (Ward et al. 2009) and the result supports the conclusion of species identification.

## Figures and Tables

**Figure 1.**
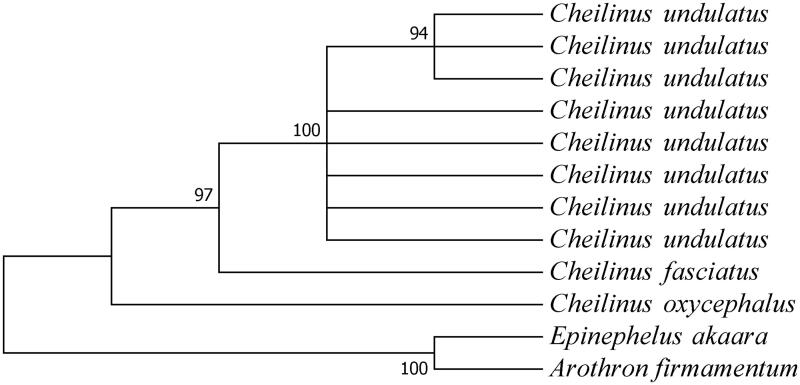
Phylogenetic relationships based on complete mtDNA sequences. Numbers at each node are neighbor-joining bootstrap proportions. The accession numbers in order are AP006742.1 (*Arothron firmamentum*), KJ700439.1 (*Epinephelus akaara*), MN399860 (*Cheilinus oxycephalus*), NC 037707.1 (*Cheilinus fasciatus*), MH688052.1 (*Cheilinus undulates*), MH675879.1 (*Cheilinus undulates*), MH688050.1 (*Cheilinus undulates*), GU296101.1 (*Cheilinus undulates*), KM461717.1 (*Cheilinus undulates*), MH688051.1 (*Cheilinus undulates*), MH675880.1 (*Cheilinus undulates*), and MH688049.1 (*Cheilinus undulates*), respectively.

## References

[CIT0001] Froese R, Pauly D 2011 FishBase. Stockholm, Sweden: FishBase; [Accessed on 3 Sup 2019]. https://www.fishbase.se/search.php.

[CIT0002] Tamura K, Stecher G, Peterson D, Filipski A, Kumar S 2013 MEGA6: Molecular Evolutionary Genetics Analysis version 6.0. Mol Biol Evol. 30:2725–2729. 24132122 10.1093/molbev/mst197 PMC3840312

[CIT0003] Ward RD, Hanner R, Hebert PD 2009 The campaign to DNA barcode all fishes, FISH-BOL. J Fish Biol. 74:329–356. 20735564 10.1111/j.1095-8649.2008.02080.x

